# Bis maltolato oxovanadium (BMOV) and ischemia/reperfusion-induced acute kidney injury in rats

**DOI:** 10.1186/2197-425X-2-3

**Published:** 2014-02-27

**Authors:** Emre Almac, Rick Bezemer, Asli Kandil, Ugur Aksu, Dan MJ Milstein, Jan Bakker, Cihan Demirci-Tansel, Can Ince

**Affiliations:** Department of Intensive Care Adults, Erasmus MC University Medical Center Rotterdam, Center Rotterdam, PO Box 2040, Rotterdam, 3000 CA The Netherlands; Department of Translational Physiology, Academic Medical Center, University of Amsterdam, Amsterdam, The Netherlands; Department of Anesthesiology, St. Antonius Hospital, Nieuwegein, The Netherlands; Department of Biology, Faculty of Science, University of Istanbul, Vezneciler, Istanbul, Turkey

**Keywords:** Acute kidney injury, Ischemia/reperfusion injury, Vanadium, Microcirculation

## Abstract

**Background:**

The aim of the present study was to test the potential protective effects of the organic vanadium salt bis (maltolato) oxovanadium (BMOV; 15 mg/kg) in the context of renal ischemia/reperfusion (30 min of ischemia) and its effects on renal oxygenation and renal function in the acute phase of reperfusion (up to 90 min post-ischemia).

**Methods:**

Ischemia was established in anesthetized and mechanically ventilated male Wistar rats by renal artery clamping. Renal microvascular and venous oxygenation were measured using phosphorimetry. Creatinine clearance rate, sodium reabsorption, and renal oxygen handling efficiency were considered markers for renal function.

**Results:**

The main findings were that BMOV did not affect the systemic and renal hemodynamic and oxygenation variables and partially protected renal sodium reabsorption.

**Conclusions:**

Pretreatment with the organic vanadium compound BMOV did not protect the kidney from I/R injury.

## Background

Acute kidney injury (AKI) is a frequent and costly clinical complication in critically ill patients. Using the RIFLE criteria in 20,126 hospitalized patients, Uchino et al. has found that 20% of the patients had some degrees of acute renal impairment and 3.7% of these patients had AKI [1]. Ischemia/reperfusion (I/R) injury occurring during surgery and shock is one of the major causes of this condition in native and transplanted kidneys [1–5]. The pathogenesis and pathophysiology of I/R-induced AKI is highly complex [4, 5]. In addition to hypoxic hit consequent to ischemia, the reperfusion phase has been associated with additional renal injury. I/R-induced activation of inflammatory pathways has been shown to worsen AKI [6–9]. Furthermore, increased production of radical oxygen species (ROS) and reactive nitrogen species (RNS) [10–13] and a regional imbalance between vasoactive mediators are believed to be of great importance in the development of AKI by leading impaired microcirculatory perfusion and to cellular damage, apoptosis, and irreversible organ failure [4, 5].

In this respect, vanadium compounds are promising in the prevention and treatment of ischemic AKI. Vanadium is an essential trace element in humans and plays various roles through different pathways in metabolism [14, 15]. Recent findings suggest that vanadium may play a pivotal role in the regulation of physiological cell growth, survival, and metabolism. Most biologically active forms of vanadium are the inorganic vanadate salt vanadyl sulfate (VOSO_4_) and the organic vanadium salt bis oxovanadium (BMOV). Especially, the organic BMOV can be used *in vitro* and *in vivo* for its beneficial regulatory metabolic roles without major side effects. BMOV and other vanadium compounds have demonstrated protective against ischemic cascades, apoptosis, and vascular endothelial dysfunction while supporting tissue repair in the heart and the brain [14–16].

In the present study, we therefore aimed to investigate the potential protective effects of BMOV in the acute phase of renal I/R and AKI. To this end, rats received 0 or 15 mg/kg BMOV intravenously 30 min before renal artery clamping, and we measured renal oxygenation and renal function up to 90 min of reperfusion.

## Methods

### Animals

All experiments in this study were approved by the Institutional Animal Experimentation Committee of the Academic Medical Center of the University of Amsterdam. Care and handling of the animals were in accordance with the EU Directive 2010/63/EU for animal experiments and guidelines for Institutional and Animal Care and Use Committees. The study has been carried out in accordance with the National Institutes of Health (NIH) Guide for the Care and Use of Laboratory Animals. Experiments were performed on 18 Wistar male rats (Harlan, the Netherlands) with mean ± SD body weight of 320 ± 30 g.

### Surgical preparation

The rats were anesthetized with an intraperitoneal injection of a mixture of 100 mg/kg ketamine (Nimatek^®^, Eurovet, Bladel, the Netherlands), 0.5 mg/kg medetomidine (Domitor, Pfizer, New York, NY, USA), and 0.05 mg/kg atropine sulfate (Centrafarm, Etten-Leur, the Netherlands). After tracheotomy, the animals were mechanically ventilated with a FiO_2_ of 0.4. Body temperature was maintained at 37°C ± 0.5°C during the entire experiment by external warming. The ventilator settings were adjusted to maintain end-tidal PCO_2_ between 30 and 35 mmHg and arterial PCO_2_ between 35 and 40 mmHg.

Vessels were cannulated with polyethylene catheters (outer diameter = 0.9 mm; Braun, Melsungen, Germany) for drug and fluid administration and hemodynamic monitoring. A catheter in the right carotid artery was connected to a pressure transducer to monitor mean arterial blood pressure (MAP) and heart rate. The right femoral artery was cannulated for blood sampling. The right femoral vein was cannulated for continuous infusion of Ringer’s lactate (15 mL/kg/h; Baxter, Utrecht, the Netherlands) and ketamine (50 mg/kg/h; Nimatek^®^, Eurovet, Bladel, the Netherlands).

The left kidney was exposed, decapsulated, and immobilized in a Lucite kidney cup (K. Effenberger, Pfaffingen, Germany) via a 4-cm incision in the left flank. Renal vessels were carefully separated under preservation of nerves and adrenal gland. A perivascular ultrasonic transit time flow probe was placed around the left renal artery (type 0.7 RB; Transonic Systems Inc., Ithaca, NY, USA) and connected to a flow meter (T206, Transonic Systems Inc.) to continuously measure renal blood flow (RBF). An estimation of the renal vascular resistance (RVR) was made as RVR [dynes/sec/cm^5^] = MAP/RBF. The left ureter was isolated, ligated, and cannulated with a polyethylene catheter for urine collection.

After the surgical protocol (approximately 60 min), one optical fiber was placed 1 mm above the decapsulated kidney and another optical fiber 1 mm above the renal vein to measure oxygenation in the renal microvasculature and renal vein, respectively, using phosphorimetry. A small piece of aluminum foil was placed on the dorsal site of the renal vein to prevent the contribution of underlying tissue to the phosphorescence signal in the venous oxygenation measurement. Oxyphor G2 (a two-layer glutamate dendrimer of tetra-(4-carboxy-phenyl) benzoporphyrin, Oxygen Enterprises Ltd., Philadelphia, PA, USA) was subsequently infused (6 mg/kg IV over 5 min) followed by a 30-min stabilization period. A short description of phosphorimetry is given below, and a more detailed description of the technology has been provided elsewhere [17–20].

### Experimental protocol

After baseline measurements were performed 30 min after Oxyphor G2 infusion, the rats were randomly assigned to one of the following groups: sham-operated time control (*n* = 6), I/R control (*n* = 6), and I/R with 15 mg/kg BMOV (*n* = 6). Considering this is the first study in which BMOV is being utilized in a model of renal I/R injury, we decided to use the recommended dosage (15 mg/kg) by CFM Pharma (Almere, the Netherlands). BMOV solutions were prepared in 2-mL isotonic saline, and infusion was initiated 30 min prior to renal ischemia at an infusion rate of 2 mL/h. Control rats received the same volume of isotonic saline without BMOV. Renal ischemia was created by 30-min clamping of the renal artery, and following the release of the clamp, measurements were continued up to 90 min of reperfusion. The experiments were terminated by infusion of 1 mL of 3 M potassium chloride (KCl).

### Blood variables

Arterial blood samples (0.5 mL) were taken from the femoral artery at baseline and 15 and 90 min after reperfusion. The blood samples were replaced by the same volume of Voluven^®^ (Fresenius Kabi Ltd., Runcom, UK). Samples were analyzed for blood gas values (ABL505 blood gas analyzer, Radiometer, Copenhagen, Denmark), hemoglobin concentration, and hemoglobin oxygen saturation (OSM3, Radiometer). Additionally, plasma creatinine concentrations were determined in all samples.

### Renal microvascular and venous oxygenation

Microvascular oxygen tension in the renal cortex (CμPO_2_), outer medulla (MμPO_2_), and renal venous oxygen tension (P_rv_O_2_) were measured by oxygen-dependent quenching of phosphorescence lifetimes of the systemically infused albumin-targeted (and therefore circulation-confined) phosphorescent dye Oxyphor G2. Oxyphor G2 (a two-layer glutamate dendrimer of tetra-(4-carboxy-phenyl) benzoporphyrin) has two excitation peaks (*λ*_excitation1_ = 440 nm, *λ*_excitation2_ = 632 nm) and one emission peak (*λ*_emission_ = 800 nm). These optical properties allow (near) simultaneous lifetime measurements in microcirculation of the kidney cortex and the outer medulla due to different optical penetration depths of the excitation light. For the measurement of renal venous PO_2_ (P_rv_O_2_), a mono-wavelength phosphorimeter was used. Oxygen measurements based on phosphorescence lifetime techniques rely on the principle that phosphorescence can be quenched by energy transfer to oxygen resulting in the shortening of the phosphorescence lifetime. A linear relationship between reciprocal phosphorescence lifetime and oxygen tension (given by the Stern-Volmer relation) allows quantitative measurement of PO_2_[17–20].

### Renal oxygen delivery and consumption

Arterial oxygen content (AOC) was calculated by (1.31 × hemoglobin × S_a_O_2_) + (0.003 × P_a_O_2_), where S_a_O_2_ is arterial oxygen saturation and P_a_O_2_ is arterial partial pressure of oxygen. Renal venous oxygen content (RVOC) was calculated as (1.31 × hemoglobin × S_rv_O_2_) + (0.003 × P_rv_O_2_), where S_rv_O_2_ is venous oxygen saturation and P_rv_O_2_ is renal vein partial pressure of oxygen (measured using phosphorimetry). Renal oxygen delivery per gram of renal tissue was calculated as DO_2_ (mL/min/g) = RBF × AOC. Renal oxygen consumption per gram of renal tissue was calculated as VO_2_ (mL/min/g) = RBF × (AOC − RVOC). The renal oxygen extraction ratio was calculated as O_2_ ER (%) = VO_2_/DO_2_ × 100.

### Renal function

For the analysis of urine volume, creatinine concentration, and sodium (Na^+^) concentration at the end of the protocol, urine samples from the left ureter were collected for 10 min. Creatinine clearance rate (CL_crea_) per gram of renal tissue was calculated with the standard formula: CL_crea_ (mL/min/g) = (*U* × *V*)/*P*, where *U* is the urine creatinine concentration, *V* is the urine volume per unit time, and *P* is the plasma creatinine concentration. Renal oxygen consumption efficiency for sodium transport (VO_2_/*T*_Na+_) was assessed as the ratio of the renal VO_2_ over the total amount of sodium reabsorbed (*T*_Na+_, [mmol/min]). *T*_Na+_ was calculated according to the following: ((CL_crea_ × *P*_Na+_) − (*U*_Na+_ × *V*)), where *P*_Na+_ is the plasma concentration of sodium.

### Data analysis

Statistical analysis was performed using GraphPad Prism version 5.0 for Windows (GraphPad Software, San Diego, CA, USA). Data are presented as mean ± SD unless otherwise stated. Statistical significance of differences between groups was tested using one-way ANOVA with Bonferroni *post hoc* tests. *P* values <0.05 were considered significant.

## Results

**Figure 1:**
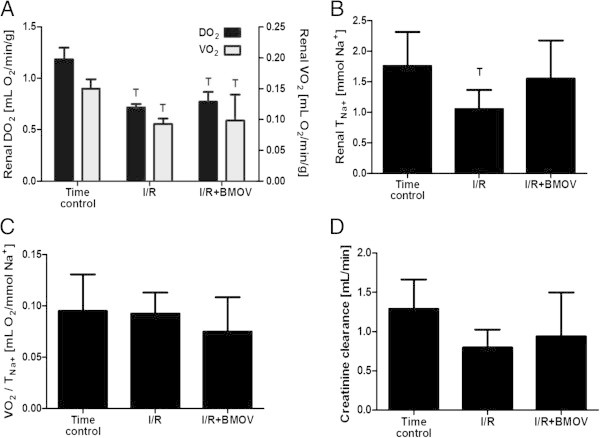
**DO**_**2**_ **and VO**_**2**_ **(A),** ***T***_**Na+**_ **(B), VO**_**2**_ **/** ***T***_**Na+**_ **(C), and CL**_**crea**_ **(D) at the end of the protocol.**

Table 1 shows the systemic and renal hemodynamic variables: MAP, RBF, RVR, DO_2_, VO_2_, CμpO_2_, and MμpO_2_ at baseline (BL), 15 min after reperfusion (R15), and 90 min after reperfusion (R90). Figure 1 shows the renal DO_2_ and VO_2_ and *T*_Na+_, renal oxygen handling efficacy (VO_2_/*T*_Na+_), and creatinine clearance rate at the end of the protocol. At baseline, there were no significant differences between groups in any of these variables.

**Table 1:** **Systemic and renal hemodynamic variables at BL, R15, and R90**

	BL (***t*** = 0 min)	R15 (***t*** = 45 min)	R90 (***t*** = 120 min)
MAP [mmHg]			
Time control	104 ± 4	101 ± 4	99 ± 4
I/R	108 ± 11	104 ± 7	90 ± 18
I/R + BMOV	108 ± 13	102 ± 24	92 ± 12
RBF [mL/min]			
Time control	4.7 ± 0.5	4.6 ± 0.6	4.6 ± 0.5
I/R	4.6 ± 1.0	2.8 ± 0.5*	3.3 ± 0.1*
I/R + BMOV	4.2 ± 1.1	2.7 ± 0.4*	3.4 ± 0.3*
RVR [dyn/s/cm^5^]			
Time control	1,777 ± 172	1,793 ± 244	1,735 ± 153
I/R	2,004 ± 672	3,097 ± 451*	2,216 ± 502*
I/R + BMOV	2,190 ± 723	3,217 ± 1,251*	2,178 ± 240*
DO_2_ [mL O_2_/min/g]			
Time control	1.21 ± 0.14	1.13 ± 0.14	1.18 ± 0.11
I/R	1.10 ± 0.24	0.65 ± 0.13*	0.72 ± 0.03*
I/R + BMOV	1.05 ± 0.32	0.64 ± 0.14*	0.77 ± 0.10*
VO_2_ [mL O_2_/min/g]			
Time control	0.14 ± 0.07	0.13 ± 0.04	0.15 ± 0.02
I/R	0.13 ± 0.10	0.10 ± 0.02*	0.09 ± 0.01*
I/R + BMOV	0.13 ± 0.07	0.11 ± 0.05	0.10 ± 0.04*
CμpO_2_ [mmHg]			
Time control	65 ± 7	62 ± 7	60 ± 6
I/R	63 ± 6	59 ± 9	54 ± 5
I/R + BMOV	66 ± 2	61 ± 9	64 ± 6**
MμpO_2_ [mmHg]			
Time control	52 ± 6	51 ± 9	49 ± 7
I/R	54 ± 5	50 ± 5	49 ± 2
I/R + BMOV	50 ± 5	50 ± 4	48 ± 3

*p < 0.05 vs time control; **p < 0.05 vs I/R.

### Systemic and renal hemodynamic and oxygenation variables

Renal I/R did not significantly affect MAP, but decreased RBF and increased RVR, which was associated with decreases in both renal DO_2_ and VO_2_. BMOV did not affect any of these macrocirculatory hemodynamic and oxygenation variables. CμpO_2_ and MμpO_2_ were similar before and after reperfusion. At the end of the protocol, CμpO_2_ was significantly higher in the BMOV treated group compared to the I/R control group.

### Renal function parameters

At the end of the protocol, renal DO_2_ and VO_2_ were decreased proportionally. Renal *T*_Na+_ was significantly reduced in the I/R control group, but in the group receiving BMOV, this decrease was not statistically significant. Renal creatinine clearance rate decreased after I/R, both with and without BMOV administration; however, it did not reach a level of significance in both groups. Similarly renal oxygen handling efficacy (VO_2_/*T*_Na+_) were maintained after I/R in both groups.

## Discussion

The aim of the present study was to test the potential protective effects of BMOV (15 mg/kg) in the acute phase of renal I/R and its effects on renal oxygenation and renal function up to 90 min post-ischemia. The main findings were that (1) BMOV did not significantly affect the systemic or the renal hemodynamic and oxygenation variables and (2) BMOV partially protected *T*_Na+_ after I/R. Furthermore, we found that microcirculatory oxygenation in the renal cortex and medulla tended to decrease after I/R. In contrast to the non-treated animals, cortical microcirculatory oxygenation was preserved in the BMOV-treated animals, but no significant differences were seen in the renal medulla.

To our knowledge, this is the first study investigating the effects of organic vanadium compound BMOV in the context of renal I/R injury. Inorganic vanadium compounds are documented to be nephrotoxic, particularly if used chronically [21], but organic vanadium compounds, such as BMOV, are known to have less side effects. In experimental studies, both pre- and post-ischemic administrations of vanadium compounds have been shown to be cytoprotective. We believe that preventive strategies are essential in order to minimize I/R injury. Therefore, we administered BMOV 30 min before the onset of ischemia. The optimal dosage of BMOV in order to prevent renal I/R injury is unknown, but administration of 15 mg/kg is recommended by the manufacturer, which is also comparable to the doses used in earlier studies [22].

Earlier studies investigating the beneficial effects of vanadium compounds after I/R injury *in vivo* have mainly been focused on the brain and on the heart. However, the type of vanadium, the type of administration, and the timing of administration varied significantly between studies. In the context of brain I/R, Kawano et al. showed in adult Mongolian gerbils that were subjected to 5-min forebrain ischemia that intraventricular injection of orthovanadate 30 min before ischemia blocked delayed neuronal death [23]. Hasegawa et al. demonstrated the neuroprotective effects of post-ischemic intraperitoneal administration of sodium orthovanadate in rats with transient middle cerebral artery occlusion 1 and 28 days after ischemia [24]. In a subsequent study, the authors determined the therapeutic time window (0, 45, and 90 min post-middle cerebral artery occlusion) and the neuroprotective dose (2 mL/kg and 12.5, 25, 37.5, and 50 mM) of sodium orthovanadate in rats [24]. Later, Shioda et al. found in a mouse model of transient middle cerebral artery occlusion that pre- and post-treatments with bis(1-oxy-2-pyridinethiolato)oxovanadium(IV) significantly reduced infarct volume in a dose-dependent manner and thereby provided neuroprotection in brain I/R injury [25]. The same group also showed that i.p. administration of bis(1-oxy-2-pyridinethiolato)oxovanadium(IV) markedly enhanced brain ischemia-induced neurogenesis in the subgranular zone of the mouse hippocampus [26]. Additionally, they found that amelioration of cognitive dysfunction following brain ischemia was positively correlated with vanadium-induced neurogenesis. Li et al. found that bisperoxovanadium attenuated cellular apoptosis in developing rat brain rescued neurons from hypoxia-ischemia brain damage [27]. Liu et al., furthermore, showed that 4 weeks of administration of sodium orthovanadate in drinking water significantly improved the outcome in rats with streptozotocin-induced diabetes after cerebral ischemia and reperfusion in terms of neurobehavioral function [28]. In the context of myocardial I/R, Geraldes et al. showed in isolated perfused rat hearts that the presence of vanadate during ischemia resulted in attenuation of acidosis and reduced lactate accumulation [29]. In anesthetized rats, Liem et al. showed that intravenous infusion of BMOV in doses of 3.3, 7.5, and 15 mg/kg i.v. decreased myocardial infarct size dose-dependently when administered before occlusion [22]. Administration of the low dose during ischemia just before reperfusion was ineffective, but administration of the higher doses was equally cardioprotective as compared with administration before occlusion. Bhuiyan et al. showed that post-ischemic treatment with bis(1-oxy-2-pyridinethiolato)oxovanadium(IV) significantly reduced infarct size and improved cardiac function in a dose-dependent manner [16]. That same group also showed that post-treatment with vanadyl sulfate significantly reduced the infarct size and significantly decreased the elevated left ventricular end-diastolic pressure, improved left ventricular developed pressure, and left ventricular contractility in a dose-dependent manner. Keyes et al. showed that the intravenous administration of bisperoxovanadium significantly reduced myocardial infarct size and improved cardiac function [30].

As demonstrated by many of the studies referenced above, vanadium compounds activate protein kinase B (Akt) signaling through inhibition of protein tyrosine phosphatases. Akt is an important signaling molecule that modulates many cellular processes such as cell growth, survival, and metabolism. Hence, by activating Akt signaling, vanadium compounds elicit cytoprotection in brain and myocardial I/R injuries. This would make vanadium also a potential candidate for reducing I/R injury in the kidney.

It must be acknowledged that our study has some limitations. Investigation of the dose-dependent response to BMOV, a longer follow-up after I/R, the acting mechanisms of BMOV, and histological evaluation of the effects of I/R and BMOV are the important limitations of our study. Since the potential benefits of BMOV in the context of I/R were never studied before, we chose to perform a relatively simple, short-term study in which we mainly focused on the effects of BMOV on renal oxygenation and function. However, longer studies are required in which different doses of BMOV are given and more detailed analysis of the involved pathways is done, together with histological evaluation. Furthermore, we used creatinine clearance rate and sodium reabsorption as measures of renal function, but we admit that these methods can lead to imprecision due to back leak phenomena and slight tubular creatinine secretion.

## Conclusions

In conclusion, pretreatment with the organic vanadium compound BMOV did not significantly affect the systemic or the renal hemodynamic and oxygenation variables and only partially protected renal sodium reabsorption after I/R. However, longer studies are required in which different doses of BMOV are given and more detailed analysis of the involved pathways is done, together with histological evaluation to fully understand the potential protective role of BMOV in the context of renal I/R.
